# Inspiring the next generation of academic physicians: the academic health careers program

**DOI:** 10.1080/10872981.2018.1530559

**Published:** 2018-10-16

**Authors:** Jennifer K. Brueckner-Collins, Terry D. Stratton, Rosemarie L. Conigliaro

**Affiliations:** a Department of Anatomical Sciences and Neurobiology, University of Louisville School of Medicine, Louisville, KY, USA; b Office of Medical Education, University of Kentucky College of Medicine, Lexington, KY, USA; c Division of General Internal Medicine, Westchester Medical Center, Valhalla, NY, USA

**Keywords:** Academic medicine, mentoring, professional development, undergraduate medical education, academic career

## Abstract

There is growing evidence in the medical education literature for the aggressive need to recruit and retain the next generation of academic physicians. In 2008, the University of Kentucky College of Medicine (UK COM) developed an academic health careers (AHCs) program for preclinical medical students as an introduction into the practice of academic medicine. The goals of this elective experience included (1) highly customized training and mentorship experiences in research, teaching, and other aspects of academic medicine; (2) information and perspectives to assist students in making informed career choices, including options for academic careers; (3) access to academic career mentors and role models related to individual faculty research interests and teaching responsibilities; and (4) opportunities to network with UK COM administrators. This short communication provides a detailed overview of the AHC experience – along with preliminary findings from a 2016–17 follow-up of program graduates exploring the program’s role in their career aspirations and decisions.

## Introduction

According to the Association of American Medical College’s annual Graduation Questionnaire (GQ), 45.0% of US medical students graduating in 2017 anticipated serving as a medical school faculty member at some point during their career, with even larger percentages indicating plans to engage in research (52.9%) or teaching (83.1%). If true, this conservatively represents roughly 6500 physicians entering the academic medical workforce in any given year, based on 2015–17 GQ data [1].

To support the future integrity of academic medicine [2], strong and deliberate faculty leadership is essential to nurturing early career interests and developing a training pipeline for potential academicians [3]. It is a concern that a lack of formal introduction to such careers during medical training [4,5] may actually impede the trajectories of those with existing interests in academic medicine or fail to spark interest among others merely exploring various career options.

To this end, many medical schools have introduced curricular threads designed to engage trainees in skills essential to success in academia, including research [6–8] and teaching [9–17]. An increasing percentage of US medical schools require formal research experiences – up to 42.8% in 2016–17 from 36.0% in 2012–13 [18]. Medical students at Vanderbilt University, for example, are afforded 3–6 months of protected research time during their M3 and M4 years, while the undergraduate training program at Duke compresses pre-clerkship training into a single year to accommodate a required, 10–12-month scholarly experience.

Formal educational training opportunities are sometimes offered among the available undergraduate coursework, though most are elective rather than required. Often, the most rigorous instructional preparation is reserved for students serving in dedicated tutor or peer mentor programs [19,20]. Typically, however, formal efforts to develop teaching skills are first encountered in residency training [15,21], though such programs vary by institution and program [22]. For all intents and purposes, the academic physician pipeline originates in residency rather than medical school [23].

While some discussion has transpired regarding differences among academic physicians – clinician-scientists, and clinician-educators – there is general agreement that modern medical practice in academic settings corresponds to a broad but definable set of competencies including but not limited to teaching, curriculum development, program evaluation, assessments, and educational leadership [24]. Few programs, however, offer courses, tracks, or experiences which approach academic medicine from this broader, comprehensive perspective, including an integrated and closely mentored overview of the skills, demands, and barriers associated with academic medical careers [3].

In 2008, the University of Kentucky College of Medicine (UK COM) developed an Academic Health Careers (AHCs) program for preclinical medical students. Modeled after a national program developed by the American Dental Education Association and the American Association of Dental Research [25,26,27], this three-credit elective provided a selected, highly motivated cohort of M1 (first-year)–M2 (second-year) students with an introduction into the practice of academic medicine. This paper describes the program and provides a preliminary follow-up summary of student participants, including the role the program may have played in their subsequent career decisions and aspirations.

## Program Description/Intervention

Beginning in the fall of 2008, applications from first year (M1) medical students were solicited for participation for the following year (M2) in a newly developed AHC elective: the stated goals of the program were to provide a small cohort of highly motivated medical students with

1. training, mentorship and experiences in research, teaching, and other aspects of an academic career;
2. information and perspectives to assist participants in making informed choices about careers, including options for academic careers;
3. access to academic career mentors and role models related to individual faculty research interests and teaching responsibilities; and
4. opportunities to network with school administrators.

The AHC program application included (1) basic demographic information, (2) two required essays detailing the student’s interest and 3–5-year career goals, (3) identification of a faculty mentor, (4) an updated CV and medical school transcript, (5) completion of a mentorship agreement form, and (5) a letter of support from the UK COM Office of Student Affairs describing the student’s academic standing and notable educational achievements – and endorsing the student’s participation in the program. This elective was restricted to students who demonstrated a high degree of motivation toward and interest in academic medicine in their application materials. The number of student participants was limited by the small team of faculty mentors who had academic protected time allocated for this type of closely mentored experience. The goal was for students to have the freedom and support to explore their specific interests in academic medicine under the coaching and guidance of an outstanding medical educator. Student participants were randomly assigned to one of the designated mentors in the program.

The program curriculum consisted of components reflecting the variety of activities and responsibilities of academic faculty. The core program requirements included the following: (1) an orientation and training session for students and mentors, (2) biweekly collaboration meetings between student and mentor, (3) faculty and academic administrator interviews, (4) a teaching practicum, (5) career reflection essays on what it means to be an academic physician, (6) a research practicum, and (7) a summative portfolio.

The orientation session consisted of a large group meeting and included all newly accepted second year medical student participants and mentors, with the goals of providing program requirements and goals. Expectations of both the mentor and student were clearly defined during this training session, along with an explicit outline of the teaching and research expectations for students in this program.

Biweekly student and mentor meetings were an integral part of the AHC curriculum. By design, this program was intended to foster close mentoring relationships between dedicated faculty advocates and medical students with a keen motivation to learn more about what academic medicine has to offer as a career. These regular meetings allowed time and space for students to develop and clearly articulate their personal objectives for the program. Students reflected on their evolving teaching philosophies over the course of the program and shared these thoughts not only in writing as essays but also verbally in conversations with their mentor. One-on-one meeting times were also used to determine teaching assignments appropriate for each student and to design and operationalize each student’s research project. In addition, these meetings were used to identify a list of faculty and administrators that each student would interview in order to gain perspective on the roles and expectations of faculty at all ranks (assistant, associate, and professor ranks) as well as insights into the responsibilities of academic deans. These interviews consisted of 1-h meetings with semi-structured interview questions, with the goal of students obtaining perspectives on specific AHCs as well as developing ease meeting with senior academic leaders and role models. The semi-structured interview protocol allowed all AHC students to touch upon similar issues, and faculty interviewees were given the questions in advance to assist them in preparing for these sessions. Students then created a synopsis of each interview and met with their mentor to discuss the insights, perspectives, and information gleaned from these interviews. All student-selected mentors were full-time mid- and senior-level faculty with major roles in students’ educational experiences (e.g., course or clerkship director, student or curriculum dean) and significant interest and experience in mentoring.

To provide the student participants with a holistic teaching experience, they were required to teach in small and large group settings, as well as provide clinical tutoring for M1 students. As student teachers, participants worked closely with their faculty mentor and with the appropriate course director to ensure their presentation met the instructional needs of the class in terms of content and style. Evaluations of these teaching experiences were conducted by the student teacher, by the student’s faculty mentor and by the M1 class being instructed. For the large group teaching requirement, each AHC student selected a discipline of particular interest to them and prepared and conducted a classroom presentation within the regular curriculum (e.g., ‘Autoimmune Pathology’ lecture in a second year course entitled Immunology, Infection and Disease). For the small group teaching requirement, AHC students were tasked with preparing and conducting a small group case conference or seminar (e.g., a journal club presentation entitled ‘A Place for Niacin in the Reduction of Cardiovascular Risk?’ as part of the Rural Physician Leadership training program). Finally, AHC student participants were also asked to serve as a tutor in a clinical setting, providing guidance as more junior M1 students worked to master various aspects of the physical exam as part of their Introduction to Clinical Medicine course.

As student participants navigated the various requirements of the AHC program, they were asked to author two career reflection essays, including one elucidating their initial impressions of what an academic physician’s job entails, as well as a summative reflection to see how their initial impressions changed over the course of the year-long program.

Each AHC student was also required to participate in a research practicum, in which they would design and execute a study exploring a medical education issue or an area of interest to them in the biomedical or clinical research arena (e.g., ‘Impact of Autoimmune Diseases on Health Status and Health Care Utilization: Cross Sectional Data from the Kentucky Women’s Health Registry’). Students participated in all aspects of the study design, preparation, and analysis to give them a sense of all domains involved in seeing a study through to its completion. All students were required to present the findings of their research in the form of a poster presentation at a local research conference.

Students were also provided a checklist to ensure completion of all components during the program. These consisted of key activities on which course assessments were based and feedback provided. Components for which no prerequisite activities existed were not required to follow a rigid sequence; however, for course administration purposes, students were encouraged to progress through these in a similar fashion.

From 2008 to 2012, a total of nine students participated completed the AHC program. In 2016, a brief online survey was conducted to solicit participants’ retrospective assessments of the program – specifically, what role, if any, it may have played in their current or future career trajectories. The study protocol was approved as exempt by the UK COM Institutional Review Board.

## Participant feedback

Of the nine AHC program ‘graduates,’ seven completed the anonymous follow-up; all were in various stages of post-graduate training or had just completed training. All respondents indicated that at least one of the teaching practicum experiences (classroom, small group, clinical) had a major positive influence on their subsequent career development.

‘I valued the classroom teaching and … definitely the interviews’ was a common sentiment among past participants. Another wrote ‘The AHC program was very helpful in opening my eyes to the intricacies of pursuing an academic medical career: Learning about research design, teaching specific topics to medical students, and putting together a portfolio.’

Additionally, all but one respondent identified the regular mentor sessions as having a positive influence: ‘Meeting with mentor regularly to discuss career goals was one of the most useful parts of the AHC program.’ Comments from respondents who found the research practicum particularly helpful suggested plans for their own future research career.

All respondents indicated that their career interests in academic medicine were either maintained or enhanced – an indication that the program had some influence. ‘I feel more informed (about careers in an academic medical center) because of AHC program,’ wrote one student. Another stated ‘…all I learned from faculty members on different rungs of the academic ladder really increased my penchant for academic medicine.’

Finally, students noted connections made with senior faculty who were later instrumental in advancing their careers as junior faculty. ‘My UK AHC experience was fantastic,’ wrote one, ‘I would not have my current career in academic medicine if I had not participated in the program.’

All respondents reported generally favorable impressions of the program and, despite the additional curricular time, believed it positively influenced their career trajectories. Suggestions for program improvements largely paralleled students’ career interests: Those planning research-directed careers suggested training in study design, research methodology, grant writing, publishing, and stronger connections with active research faculty. In contrast, those geared more towards education identified the need for further training in curriculum design, test-item writing, and teaching skills. Further suggestions included broader information on the various academic medicine career paths, contract negotiation skills, and the financial aspects of working in an academic institution.

## Discussion

Our AHC program was designed to address many of the recommendations found in the literature for encouraging undergraduate medical student engagement in academic medicine, including the opportunity to design, conduct and present an educational research project, engage in an academic mentoring relationship, and explore the multifaceted nature of academic jobs. Although our students assessed the program in an overall positive light, it is likely too early to document career outcomes or whether the AHC program may have influenced that choice.

While the goal of this paper was not to explicitly assess the program’s impact, it is worth noting some potential caveats. First, given that this program was an elective, the participant feedback may reflect a prior interest in this experience and thus, some potential value in it. If students’ plans for possible academic medical careers motivated their interest in the program, enrollment may not truly have captured the less interested or casually curious.

Second, the curricular timing or placement of the AHC elective may have limited students’ participation. Although not mentioned explicitly, it is possible that such an experience might be more popular and beneficial in the fourth year, as students approach their residency training and are thinking more about their future careers.

Third, based on student feedback, it may be that a ‘core’ curriculum could provide a nucleus around which to target learner-specific career goals, with tracking into clinician-educator or clinician-scientist paths, and specific training and skills geared toward these career options. A needs assessment of potential participants might allow the targeting of particular skills and competencies.

Fourth, the generalizability of our experience in creating and offering the AHC program elective is not entirely clear. Still, as a midsized but growing academic medical center, the opportunities at UK COM for providing students with the prescribed experiences are probably within the reach of similar institutions. Since faculty time, and not financial support, is the major consideration in providing a comparable academic medical training experience, doing so is not overly prohibitive. Indeed, the logistics of mentor recruitment, identification of practical training opportunities, and accommodation for some level of individual focus or self-direction are among the more challenging aspects. The need for faculty champions for this type of endeavor is critical; in this case, the departure of key faculty mentors limited the duration of the program from 2008 to 2012. Recruitment of adequate faculty mentors is vital to the longevity of such programs.

Admittedly, students’ general educational experience – including the learning environment and the quality of teachers and mentors they encounter – is perhaps as likely as any elective to influence career interests in this type of practice setting. Even so, supplementing these more idiosyncratic influences with a focused, dedicated training experience might, if well placed, provide the added motivation for students to embark on academic medical careers well in advance of their first faculty position.

## Conclusion

We have described an undergraduate medical elective experience designed to introduce students to academic medicine and key aspects of this career path. This paper outlines the curricular structure and content, along with preliminary feedback from past participants regarding the program’s required components and their role in guiding future career interests and plans. Especially for training programs interested in developing early recruiting pipelines for future graduates, the implementation of similar programs may help to enhance knowledge of and interest in academic medicine as a career trajectory.
